# 

**Published:** 1900-08-15

**Authors:** 


### ANNOUNCEMENTS.

### THE MICHIGAN STATE BOARD OF EXAMINERS.

The next meeting of this Board will be held in the city of Detroit, at the Hotel Cadillac, October 9th, at 9 A M. Candidates for registration should present themselves at the first session.

FRANK O. GILBERT, D.D.S., *Sec'y.*,
Bay City, Michigan.

AT the seventeenth annual session of the National Association of Dental Examiners, held at Old Point Comfort, Va, July 13th and 14th, the following officers were elected for the ensuing year: President, V. J. Turner, Raleigh, N. C.; Vice-President, J. F. Dowsley, Boston, Mass.; Secretary and Treasurer, Charles A. Meeker, Newark, N. J.

Committee on Colleges: C. C. Chittenden, Madison, Wis; J. A. Hall, Collinsville, Ala.; M. F. Finley, District of Columbia.

Dr. Dowsley and Dr. Meeker were appointed to represent the Association at the Paris Congress.

